# Cost-utility and cost-effectiveness of SELFIE, a transdiagnostic ecological momentary intervention for improving self-esteem in youth aged 12–26 years exposed to childhood adversity: Findings from a multicenter randomized controlled trial

**DOI:** 10.1017/S003329172610395X

**Published:** 2026-04-13

**Authors:** Anouk Boonstra, Maud Daemen, Sander Osstyn, Ron Handels, Ruben Drost, Mary Rose Postma, Iris Hoes-van der Meulen, Nele Volbragt, Dorien Nieman, Philippe Delespaul, Marieke van der Pluijm, Josefien Johanna Froukje Breedvelt, Mark van der Gaag, Ramon Lindauer, Jan Boehnke, David van den Berg, Lieuwe de Haan, Silvia Evers, Filip Smit, Claudi Bockting, Therese van Amelsvoort, Ulrich Reininghaus

**Affiliations:** 1 Department of Psychiatry and Neuropsychology, Mental Health and Neuroscience (MHeNs) Research Institute, Faculty of Health, Medicine and Life Sciences (FHML), Maastricht University, Maastricht, The Netherlands; 2 Central Institute of Mental Health, Department of Public Mental Health, Medical Faculty Mannheim, University of Heidelberg, Mannheim, Germany; 3 Alzheimer Centre Limburg, Faculty of Health Medicine and Life Sciences, Mental Health and Neuroscience Research Institute, Department of Psychiatry and Neuropsychology, Maastricht University, Maastricht, The Netherlands; 4 Department of Health Services Research, Care and Public Health Research Institute (CAPHRI), Faculty of Health, Medicine and Life Sciences (FHML), Maastricht University, Maastricht, The Netherlands; 5 Mondriaan Mental Healthcare Centre, Heerlen, The Netherlands; 6 Prodeba Mental Health Care, Leiden, The Netherlands; 7 Department of Psychiatry, Amsterdam University Medical Centre (location AMC), Amsterdam Public Health, Amsterdam, The Netherlands; 8 Centre for Urban Mental Health, University of Amsterdam, Amsterdam, The Netherlands; 9 Department of Child and Adolescent Psychiatry, Institute of Psychiatry, Psychology and Neuroscience, King’s College London, London, United Kingdom; 10 Department of Clinical Psychology, Vrije Universiteit, Amsterdam, The Netherlands; 11 Department of Child and Adolescent Psychiatry, Amsterdam UMC (location AMC), Amsterdam, The Netherlands; 12 Levvel, Academic Centre for Child and Adolescent Psychiatry, Amsterdam, The Netherlands; 13 School of Health Sciences, University of Dundee, Dundee, United Kingdom; 14 Department of Psychosis research, Parnassia Psychiatric Institute, The Hague, the Netherlands; 15 Department of Epidemiology and Biostatistics, Amsterdam University Medical Centers (location VUmc), Amsterdam, The Netherlands; 16 Health Service and Population Research Department, Institute of Psychiatry, Psychology & Neuroscience, King’s College London, London, UK

**Keywords:** childhood adversity, cost-effectiveness, cost-utility, ecological Momentary Intervention, self-esteem

## Abstract

**Background:**

A novel transdiagnostic blended Ecological Momentary Intervention (EMI) for improving self-esteem in youth who experienced childhood adversity has shown beneficial effects. However, evidence on the cost-effectiveness of SELFIE and EMIs overall is lacking.

**Methods:**

In this RCT-based economic evaluation, participants aged 12–26 years with low self-esteem (<26 on the Rosenberg Self-Esteem Scale [RSES]) and experienced childhood adversity were recruited from specialized mental health services and the general population and randomly allocated to CAU (control condition) or CAU + SELFIE (experimental condition). The Trimbos/iMTA Questionnaire for Costs Associated with Psychiatric Illness (TiC-P), the RSES, and EQ-5D-5L were assessed at baseline, post-intervention, and 6-, 18-, and 24-month follow-up. Incremental cost-effectiveness (ICER) and cost-utility (ICUR) ratios, and acceptability curves, were generated. Sensitivity and subgroup analyses assessed robustness.

**Results:**

From a societal perspective, the ICER presented €1,219 per improved point of self-esteem, and the ICUR presented €53,986 per QALY gained. The probability of cost-effectiveness was 26% at a willingness-to-pay (WTP) threshold of €20,000/QALY and 49% at €50,000/QALY. At €50,000/QALY, the probability of cost-effectiveness was 77% from a healthcare perspective (ICUR €15,671/QALY gained) and 87% for participants recruited from specialized mental health services (ICUR €–14,567/QALY gained).

**Conclusions:**

At the societal level, the SELFIE intervention exceeded the WTP threshold. Tests for robustness showed higher probabilities of cost-effectiveness from a healthcare perspective, likely reflecting the absence of educational productivity loss costs in the societal perspective, and in youth recruited from specialized mental health services. The innovative field of blended EMIs yields encouraging first results, urging more cost-effectiveness research.

## Introduction

Childhood adversity (i.e., abuse, neglect, bullying and/or parental conflict) is a major risk factor for the development of mental disorders (Aas et al., 2016; Copeland et al., 2018; McKay et al., 2020) and is prospectively linked to higher societal costs (Thielen et al., 2016). As mental disorders substantially contribute to global disease burden (GBD 2019 Mental Disorders Collaborators, 2022), cost-effective interventions are direly needed (Knapp & Wong, 2020). On the pathway from childhood adversity to adult mental ill-health, self-esteem has been identified in Ecological Momentary Assessment (EMA) as a candidate momentary mechanism that can be targeted using Ecological Momentary Intervention (EMI) (Daemen et al., 2023; Mazereel et al., 2024). EMAs and EMIs are built on the notion that experiences and behaviors are both occurring and best transformable in daily life (Schulte-Strathaus et al., 2022), where EMIs operate as a ‘therapist in the pocket’ that responds with personalized support so users can manage their own mental health in real time. These innovative mobile applications are now applied, for example, in third-wave behavioral therapy interventions, with studies showing feasibility, efficacy (van Aubel et al., 2020), and medium-to-large effect sizes (Rauschenberg et al., 2021). Moreover, EMIs might be especially suited and accessible to aid young people, for whom digital life is inherent to daily life (Odgers & Jensen, 2020).

A novel transdiagnostic blended Ecological Momentary Intervention (EMI) (i.e. SELFIE) was designed to target the dynamic construct of self-esteem in daily life in young people aged 12–26 years with prior exposure to childhood adversity and low self-esteem (Daemen et al., 2021; Reininghaus et al., 2023). SELFIE consists of three face-to-face sessions, three e-mail contacts, and an EMI delivered via a smartphone-based application over 6 weeks. The efficacy of SELFIE on self-esteem has previously been demonstrated in a 2-arm, parallel-group, assessor-blinded, randomized controlled trial (RCT) (Reininghaus et al., 2023). Effects on self-esteem were sustained at 18- and 12-month follow-up, and effect sizes were small to moderate on self-esteem (Cohen *d* = .54) and secondary outcomes (Cohen *d* = .20–.53) across post-intervention and follow-up (Reinhold et al., 2025), but evidence on the cost-effectiveness and cost-utility of SELFIE is lacking.

Health-economic evaluations of interventions for youth exposed to childhood adversity are needed to provide indications about the benefits and costs of investment in (digital) mental health interventions, but are scant (Steptoe et al., 2019) and, to our knowledge, nonexistent for EMIs in youth. One study so far has provided a health-economic evaluation of an “experience sampling method intervention” (ESM-i), but this was an ESM-i for depression among adults (Simons et al., 2017). In youth with prior exposure to childhood adversity, health-economic reviews of interventions have primarily identified studies focusing on specific types of adversity or on post-traumatic stress disorder (PTSD). Moreover, no study has investigated the cost-effectiveness of such interventions targeting young people aged 12–25 years, as inclusions were either under or above the age of 18 years (Spencer et al., 2023; von der Warth, Dams, Grochtdreis, & König, 2020). Importantly, youth mental health interventions must be studied throughout young adulthood, as the split in care at the age of 18 years that exists in many countries produces harmful interruption or termination of care for many young people amid this important developmental period in life (Boonstra et al., 2024; Rosina et al., 2024). Limited access to care is a significant problem for young people (Leijdesdorff et al., 2021), also among youth who experienced childhood adversity (Schweer-Collins & Lanier, 2021; Soneson et al., 2024), where digital and blended interventions are well placed to address this issue, improve accessibility, and drive service innovation (McGorry et al., 2022; McGorry et al., 2024). In the present study, we aimed to investigate the cost-effectiveness and cost-utility of SELFIE, a blended EMI for improving self-esteem among help-seeking youth with low self-esteem who experienced childhood adversity in addition to care as usual (CAU), in comparison with CAU only, from a societal perspective.

## Methods

### Study design

The current 2-arm, parallel-group, assessor-blinded RCT examined the cost-effectiveness and cost-utility of SELFIE as described in our study protocol (Daemen et al., 2021). The economic evaluation applied a societal perspective over a 24-month follow-up period. After completion of baseline assessment, participants were randomly allocated to one of two parallel conditions: SELFIE in addition to CAU (experimental condition) or CAU only (control condition). Recruitment lasted from January 2019 to June 2021 from specialized mental health services (that is for severe, complex, or long-term psychiatric conditions) in three regions in the Netherlands (i.e., Noord-Holland (Amsterdam University Medical Centre’s Location AMC; Levvel), Zuid-Holland (Prodeba; Parnassia Group), and Limburg (Mondriaan; Koraalgroep; Lionarons GGZ)). Participants from these services were recruited primarily via clinicians and flyers were put in the services. Furthermore, participants were recruited from the Dutch general population using flyers and (social media) adverts. We used a computer-generated sequence, with block randomization in blocks of 6, stratified by region of specialized mental health services of collaborating centers (i.e., Noord-Holland, Zuid-Holland, Limburg) or as external admission (i.e., from the Dutch general population).

The SELFIE trial has been registered (ID: NL-OMON52597) and has been approved by the Medical Ethics Review Committee (MERC) at Maastricht University Medical Centre (MUMC+), the Netherlands (reference: NL64393.068.17). The present study followed the Consolidated Health Economic Evaluation Reporting Standards (CHEERS) (Husereau et al., 2022a; Husereau et al., 2022b, Supplementary Table 2).

### Participants

The inclusion criteria of the participants were as follows: (1) aged 12–26 years, (2) exposure to childhood adversity, (3) below-average self-esteem (score < 26) as measured with the Rosenberg Self-Esteem Scale (RSES) (Rosenberg, 1965), (4) willingness to take part in the study, (5) ability to provide informed consent, and (6) parental consent if the participant is a minor (<18 years). Exclusion criteria were: (1) insufficient command of Dutch, and/or (2) psychiatric symptoms due to an organic cause (e.g. brain injuries) (Daemen et al., 2021; Reininghaus et al., 2023).

### Experimental condition and control condition

The control condition, CAU, was defined as having access to any form of standard health care and/or social services. This could also mean having no form of care or starting or stopping care over time, in the way participants would have done without inclusion in this study. During the intervention period, none of the participants received manualized treatments that predominantly targeted self-esteem (e.g. Competitive Memory Training (COMET)).

The experimental condition comprised the manualized SELFIE intervention in addition to CAU. This 6-week blended intervention for improving self-esteem entailed three face-to-face sessions of one hour (on-site or online) and three email contacts delivered by 18 trained mental health professionals, as well as an EMI administered through the smartphone-based PsyMate® application, all based on the principles of EMIs (Heron & Smyth, 2010; Myin-Germeys et al., 2016; Reininghaus, 2018; Reininghaus, Depp, & Myin-Germeys, 2016) and a guided self-help approach based on cognitive behavioral therapy (De Neef, 2016). The EMI translated the training from the face-to-face sessions into young people’s daily lives based on three types of delivery schemes (detailed in Daemen et al., 2021; Reininghaus et al., 2023). Besides introducing new elements (that is principles and techniques to be continued by the participant by use of the EMI), the contacts with the SELFIE therapists provided a chance to reflect on progress and discuss potential issues experienced with the EMI.

### Valuation of outcomes and costs

Outcome measures to capture health-economic outcomes, health-related quality of life (HRQoL), and resource use were examined at baseline, post-intervention, and at 6-, 18-, and 24-month follow-up.

The outcome measure used in the **cost-effectiveness analysis (CEA)** was the RSES (Rosenberg, 1965), a well-established self-report measure of global self-esteem with psychometrically sound internal reliability and structural validity (Schmitt & Allik, 2005). Among young people, sufficient evidence was found for the RSES’s structural validity and internal consistency, and it may even be considered a gold standard (Greco et al., 2025). The Dutch RSES has also shown good validity and high internal consistency (Everaert, Koster, Schacht, & De Raedt, 2010; Franck, De Raedt, Barbez, & Rosseel, 2008). The RSES contains 10 items, each rated on a 4-point Likert scale, where 0 = “strongly disagree” and 3 = “strongly agree.” The total score ranges between 0 and 30, with higher total scores indicating higher self-esteem.

The outcome measure used in the **cost-utility analysis (CUA)** was the EQ-5D-5L to assess HRQoL (EuroQol Research Foundation, 2019). The EQ-5D-5L has satisfactory validity (Janssen et al., 2013) and reliability (Feng, Kohlmann, Janssen, & Buchholz, 2021) and is the recommended scale for health-economic evaluations in the Netherlands (Hakkaart-van Roijen et al., 2024a). This self-report measure consists of five dimensions (*mobility, self-care, usual activities, pain/discomfort* and *anxiety/depression*). The level for each dimension is entered in a sequence for the five dimensions, called a health state, later converted to Quality-Adjusted Life Years (QALYs) as detailed in the Analysis section under Utility calculations.

Resource use data were gathered from a societal perspective, using the Trimbos/iMTA Questionnaire for Costs associated with Psychiatric Illness (TiC-P) (Hakkaart-van Roijen, Straten, Tiemens, & Donker, 2002). The TiC-P is a feasible and reliable questionnaire for recording frequency of service use (healthcare costs and emergency care costs) and productivity loss (days of illness at work and days to be compensated) (Bouwmans et al., 2013). The specific services under the main categories of healthcare and emergency care are specified in Table 1. The last main category, medication use, was inquired about as part of multiple standardized interviews for the RCT, specifying medication name and quantity of use. In the Netherlands, basic health insurance is mandatory and covers this care.

**Table 1.** Unit prices of societal costs, each corrected to August 2023 for inflation

**Cost category^a^**	Unit	Data source	Original price year	Inflation-corrected cost per unit, 2023 (€)^b^
**Healthcare costs**				
General practitioner	Contact	Hakkaart-van Roijen et al. (2024a)	2022	32
POH-GGZ	Contact	Hakkaart-van Roijen et al. (2024a)	2022	21
Psychologist^a^	Contact	Hakkaart-van Roijen et al. (2024a)	2022	112
Dietician	Contact	Hakkaart-van Roijen et al. (2024a)	2022	25
Healthcare social worker	Contact	Hakkaart-van Roijen et al. (2024a)	2022	130
Psychiatrist^a^	Contact	Hakkaart-van Roijen et al. (2024a)	2022	140
Substance use services^a^	Contact	Hakkaart-van Roijen et al. (2024a)	2022	179
Alternative care^a^	Contact	Hakkaart-van Roijen et al. (2024a)	2022	51
Semi-mural care	Hour	Hakkaart-van Roijen, Van der Linden, Bouwmans, Kanters, and Tan (2015)	2014	17
**Emergency care costs**				
Police at the door	Hour	Statistics Netherlands (2022)	2020	58
Police transport to facility	Hour	Statistics Netherlands (2022)	2020	58
Ambulance	Transport	Hakkaart-van Roijen et al. (2024a)	2022	671
Crisis service^a^	Contact	Hakkaart-van Roijen et al. (2024a)	2022	205
Emergency room (ER)	Contact	Hakkaart-van Roijen et al. (2024a)	2022	263
Psychiatric hospital	Day	Hakkaart-van Roijen et al. (2024a)	2022	334
**Medication costs^c^**		https://www.medicijnkosten.nl/	2023	Various
**Productivity loss costs**	Work hour	Hakkaart-van Roijen et al. (2024a)	2022	41

*Abbreviation:* POH-GGZ = general practitioner assistant in mental health care.

a Due to differences in categories, the data source categories were matched to the following TIC-P categories: (1) TIC-P “psychologist” was matched with the following data source categories: average of the costs of both an independent care provider and a generalist basic mental healthcare consultation; (2) TIC-P “psychiatrist” was matched with: average of the costs of various specialist mental healthcare consultations; (3) TIC-P “substance use services” was matched with: average of the costs of several specialist mental healthcare consultation, psychiatric institution (day care), and independent care provider in the basic mental healthcare consultation; (4) TIC-P “alternative care” was matched with: average of the costs of all paramedical care categories (physiotherapist, speech therapist, remedial therapist, occupational therapist, dietician and combined lifestyle interventions); and (5) TIC-P “crisis service” was matched with: average of the costs of various specialist mental healthcare consultations and staying at a psychiatric institution (day care).

b The used inflation factors were *1.0209 for cost data from 2022 (Hakkaart-van Roijen et al., 2024b), *1.2380 for semi-mural care costs from 2014 (Statistics Netherlands, 2024) and *1.2044 for costs for police at the door and a police transport to a facility from 2020 (Statistics Netherlands, 2022).

c Medication costs could not be defined with a cost per unit or listed independently, as over 100 types of medication were used with varying doses and frequencies.

**Table 2.** Baseline characteristics of included participants

Variable	*n*	Experimental condition CAU + SELFIE (*n* = 85)	*n*	Control condition CAU (*n* = 89)	*N*	Full sample (*N* * *= 174)
**Demographic variables**						
Age (years), mean (SD)	85	20.9 (3.0)	89	20.5 (3.2)	174	20.7 (3.1)
Gender, n (%)^a^	85		89		174	
Female		73 (86%)		81 (91%)		154 (89%)
Male		11 (13%)		8 (9%)		19 (11%)
Nonbinary		1 (1%)		0 (0%)		1 (1%)
Level of education (completed, ongoing), *n* (%)^b^^,^^c^	85		88		173	
Low		42 (49%)		35 (40%)		77 (45%)
Middle		34 (40%)		41 (47%)		75 (43%)
High		9 (11%)		12 (14%)		21 (12%)
Self-assigned ethnicity, n (%)	85		89		174	
White majority		73 (86%)		76 (85%)		149 (86%)
Migrant/ethnic minority		12 (14%)		13 (15%)		25 (14%)
Medication use, n (%)	85	52 (61%)	89	60 (67%)	174	112 (64%)
Study center/recruitment route, n (%)	85		89		174	
Noord-Holland		6 (7%)		6 (7%)		12 (7%)
Zuid-Holland		11 (13%)		14 (16%)		25 (14%)
Limburg		26 (31%)		24 (27%)		50 (29%)
General population		42 (49%)		45 (51%)		87 (50%)
**Childhood adversity, mean (SD)**						
Childhood trauma (CTQ)	85		89		174	
Emotional abuse		14.1 (5.0)		14.8 (5.1)		14.5 (5.1)
Physical abuse		6.8 (3.1)		7.5 (3.4)		7.2 (3.3)
Sexual abuse		8.2 (5.0)		10.2 (7.0)		9.2 (6.2)
Emotional neglect		14.5 (4.7)		14.6 (5.0)		14.6 (4.9)
Physical neglect		8.8 (3.4)		8.5 (2.0)		8.7 (3.1)
Bullying (RBQ)	82		74		156	
Frequency		3.0 (1.0)		3.1 (1.1)		3.07 (1.1)
Severity		3.0 (1.1)		3.0 (1.0)		3.02 (1.0)
Parental conflict (CECA)	84		89		173	
Frequency		2.1 (1.8)		2.2 (1.7)		2.2 (1.7)
Severity		1.7 (1.6)		2.0 (1.5)		1.9 (1.5)
**Self-esteem** (RSES)**, mean (SD)**	85	18.5 (3.9)	89	19.4 (3.7)	174	18.9 (3.8)
**Quality of Life** (EQ–5D–5L)**, mean (SD)**	85	0.63 (0.20)	89	0.58 (0.23)	174	0.60 (0.22)

*Abbreviations:* CAU = care as usual; CECA = Childhood Experience of Care and Abuse Interview; CTQ = Childhood Trauma Questionnaire; SD = standard deviation; RBQ = Retrospective Bullying Questionnaire; RSES = Rosenberg Self-esteem Scale; EQ-5D-5L = EuroQol-5 Dimension Five Levels.

a The number of participants identifying as nonbinary was too small to report numbers without compromising identifiability.

b Education degree adapted from the Dutch Standard Classification of Education (Statistics Netherlands, 2019).

c Data missing for one participant in CAU.

### Other measures

Types of childhood adversity were measured with the following self-report measures: first, the Childhood Trauma Questionnaire (CTQ) was used to measure exposure to physical abuse, emotional abuse, sexual abuse, physical neglect, and emotional neglect in childhood. The Retrospective Bullying Questionnaire (RBQ) was used to measure past physical, verbal, and indirect bullying. Part of the Childhood Experience of Care and Abuse (CECA) Interview was used to measure parental conflict.

### Analyses

All analyses were performed using STATA (Version 17.0, Standard Edition). Data were prepared and analyzed according to the intention-to-treat principle. This health-economic evaluation was conducted from a societal perspective.

#### Cost calculations

All costs in the present study were measured in Euro (€) and consisted of the following categories: healthcare costs, emergency care costs, medication costs, and productivity loss costs. In Table 1, the inflation-corrected unit prices of societal costs are shown, along with their respective data sources, and the composition of the cost categories is provided in the footnote. Most unit prices were available from Hakkaart-van Roijen et al. (2024b), and the rest were derived from Statistics Netherlands (2022) and Hakkaart-van Roijen et al. (2015). Each resulting unit cost was multiplied by the frequency of resource use that each participant reported in the TiC-P. Units (e.g. days or hours) were matched between the TiC-P and costing resources, assuming 12 hours per day for semi-mural care. Intervention costs were calculated by adding up estimated one-off costs and participation costs of the digital application and the therapist contacts, as detailed in Supplementary File 1.

Next, the calculated costs were prepared for the CEA and CUA. As resource utilization was recalled for the past 4 weeks at each measurement point, the area under the curve method was used to cover the interval between measurements. Usage in the final period was adjusted to ensure a total follow-up of exactly 26 months from baseline, providing a consistent time horizon across participants.

#### Utility calculations

For the utility part of the CUA, the HRQoL health states of the EQ-5D-5L were first converted into utilities using the Dutch tariff (Versteegh et al., 2016). A utility is a value that typically ranges from 0 (meaning death) to 1 (meaning perfect health) and can be below 0 (worse than death). Next, the utilities were converted into QALYs by multiplying the utilities of the health states by the time between two measurement moments and summed over the full follow-up period of the trial using the area under the curve method (Manca et al., 2005).

#### Main analyses

Missing data were handled following the guidance described in Faria, Gomes, Epstein, and White (2014). Missing data were inspected, classified as missing-at-random, and handled by use of multiple imputation with 20 imputations on the end-point level in terms of QALYs, RSES, and four cost categories (medication use, productivity loss, healthcare use, and emergency care use).

Next, data were analyzed over a 26-month time horizon: the time from baseline measure to 24-month post-intervention follow-up. The intervention lasted 6 weeks, with an additional week of planning beforehand and a week afterwards, totaling 8 weeks or 2 months, added to the 24 months of post-intervention measurements. Because the follow-up period exceeded 1 year, costs and outcomes accruing in the second year of follow-up were discounted, applying annual discount rates of 4.0% for costs and 1.5% for QALYs and RSES (Hakkaart-van Roijen et al., 2015). Uncertainty was addressed using bootstrapping with 1,000 replications performed after the multiple imputation process. Within each bootstrap replication, incremental outcomes (total costs, RSES, and QALYs) were estimated using linear mixed-effects regression models with baseline adjustment. Gaussian distributions with identity link functions were specified, with outcome modeled as the dependent variable and trial arm and the corresponding baseline value included as independent variables. Incremental differences between groups and their 95% bootstrap intervals were derived from the bootstrap. All details are described in Supplementary File 2, based on reporting guidelines by van Buuren (2018).

An incremental cost-effectiveness ratio (ICER; incremental costs divided by incremental effects) and incremental cost-utility ratio (ICUR; incremental costs divided by incremental QALYs) were calculated, and an incremental cost-utility plane (ICUP) and cost-effectiveness plane (ICEP) were generated. The probability of the intervention being cost-effective compared to CAU was displayed in a cost-utility acceptability curve (CUAC) for QALYs relative to a range of willingness-to-pay (WTP) thresholds, and in a cost-effectiveness acceptability curve (CEAC) for effects (RSES). The probabilities of cost-effectiveness based on QALYs, and thus the CUAC, are leading, as QALYs are the empirically established standard for health-economic analyses.

For valuation of the cost-utility outcomes, QALYs were monetized with a WTP for each QALY gained, and the literature was inspected to find appropriate WTP thresholds. Although not conducted beyond the age of 18 years, cost-effectiveness studies for youth who experienced childhood adversity used thresholds between 20,000 and 50,000 pounds (Mavranezouli et al., 2020a; Shearer et al., 2018) or Australian dollars (Mihalopoulos et al., 2015). Furthermore, a WTP of €50,000 was used in the only slightly comparable study to the present one, namely ESM-i for depression among adults in the Netherlands (Simons et al., 2017). In the Netherlands, a WTP threshold of €20,000/QALY is advised in case of treatment for mild depression, and €50,000/QALY for moderate to severe levels of depression (Vijgen et al., 2018). Based on these resources, WTP thresholds of €20,000/QALY and €50,000/QALY were used in the analysis of the present health-economic evaluation.

#### Sensitivity and subgroup analyses

Sensitivity analyses were conducted identical to the main analysis but (1) excluded productivity loss costs to obtain a healthcare-only perspective, and (2) excluded one-off intervention costs (e.g. therapist training, developer costs for implementation, and server rent) to learn about cost-effectiveness if the already developed SELFIE application would be used routinely (i.e. in a steady-state healthcare system). In addition, (3) a complete case analysis was adopted, excluding the participants with missing values.

Given the different recruitment methods, forms of childhood adversity, and treatment statuses, subgroup analyses were conducted. It was expected that the probability of cost-effectiveness might be higher for participants recruited from the general population, as they applied for the intervention of their own motivation instead of participation being proposed by a clinician. Furthermore, the probability of cost-effectiveness might be higher for participants who were already in treatment during enrollment in this study due to the additional effects of other treatments that benefited their (mental) well-being, care needs, and productivity. Next, working with each form of childhood adversity was central to this study, and no differences in effects were expected to be found. Hence, we aimed to examine each type of childhood adversity in terms of cost-effectiveness between CAU + SELFIE and CAU. These types were established using the moderate–severe cutoff scores determined by Bernstein and Fink (1998) for the CTQ: ≥13 for emotional abuse; ≥10 for physical abuse; ≥15 for emotional neglect; ≥10 for physical neglect; and ≥8 for sexual abuse. For parental conflict (CECA: Bifulco, Brown, & Harris, 1994), the cutoff was at ≥3 for both frequency and severity. For bullying (RBQ: Schäfer et al., 2004), scores were dichotomized with a cutoff of ≥3 for frequency and ≥4 for severity. All sensitivity analyses and subgroups were predetermined before accessing the data for the health-economic evaluation.

## Results

### Participant characteristics, completion rates, and cost descriptives

The mean age of the full sample was 20.7 years (SD = 3.1). Most participants were female (*n =* 154, 89%). The majority had a self-ascribed White ethnicity (*n* = 149, 86%) and used medication (*n* = 112, 64%). Further baseline characteristics and scores on the CTQ, RBQ, and CECA (for childhood adversity), RSES (for self-esteem), and EQ-5D-5L (for HRQoL) are shown in Table 2. Other clinical characteristics of questionnaires not used in the present study are detailed elsewhere (Reininghaus et al., 2023).

**Figure 1.**
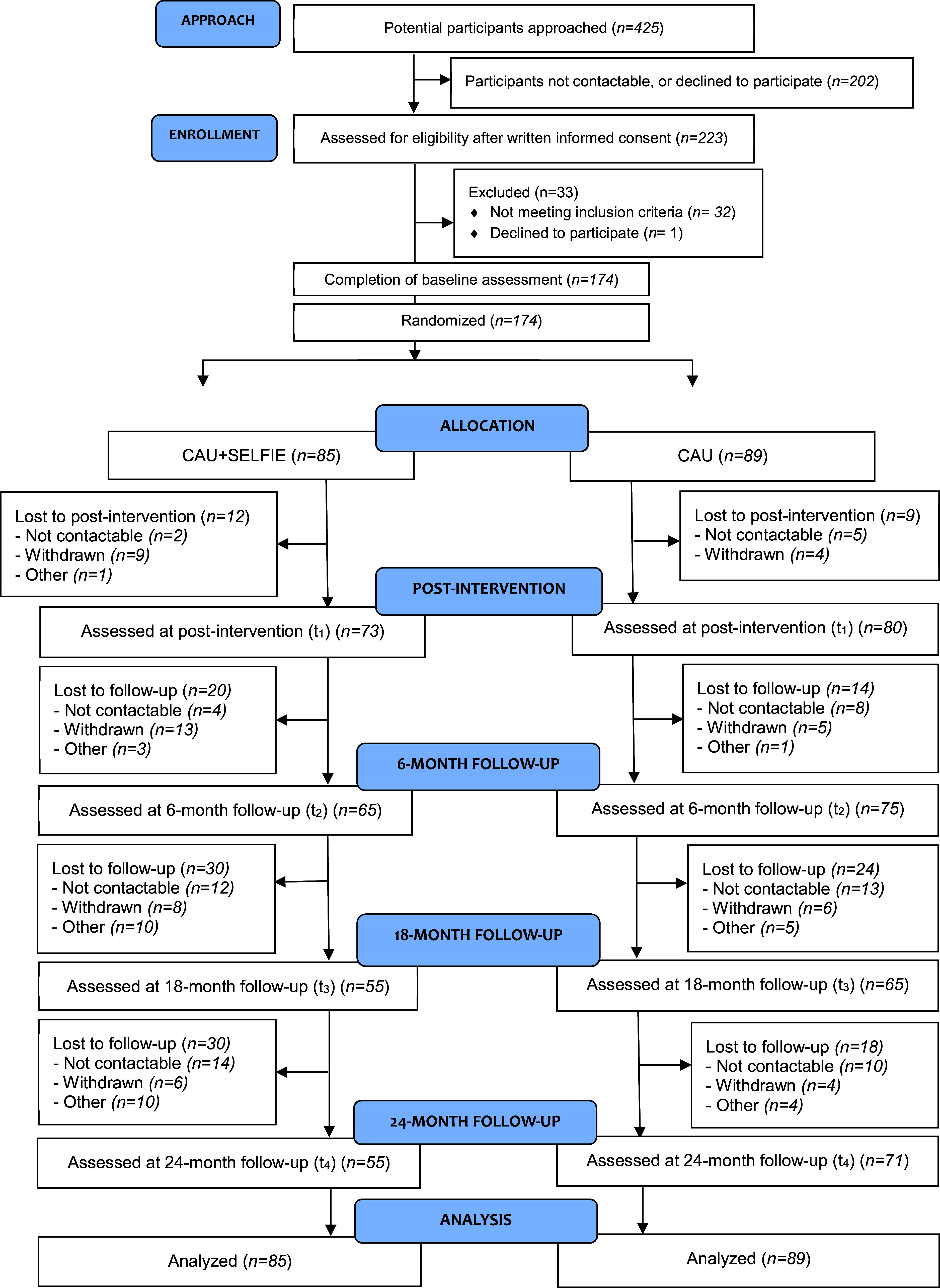
Flowchart of the SELFIE study (using the CONSORT checklist (Montgomery et al., 2018))

From a total of 174 participants, random allocation resulted in 85 participants randomized to the CAU + SELFIE arm and 89 participants to the CAU arm, as shown in Figure 1. As for completion of measures per time point: 100% of data were complete for all measures at baseline (see Supplementary Table 1). For education level and TIC-P, data were missing for one person, resulting in 99% completeness of data. The post-intervention completeness rate was 89% for the RSES, 87% for the TiC-P and EQ-5D-5L, and 85% for the medication checklist. At T4, this was 73%, 71%, and 70%, respectively.

The post-imputation average outcomes for utilities, self-esteem, and the cost categories are displayed per time in Table 3. The sum of the inflation-corrected intervention costs was €14,781 for one-off costs and €38,384 for participation costs, totaling €53,164 (*N* = 174) (see Supplementary File 1).

**Table 3.** Average outcomes for utility, RSES and cost categories after imputation of missing data (*N* = 174), mean (SD)

	T0 (baseline)			T1 (post-intervention)			T2 (6 months)			T3 (18 months)			T4 (24 months)			Total follow-up period (0-26 m)	
	CAU +SELFIE	CAU		CAU +SELFIE	CAU		CAU +SELFIE	CAU		CAU +SELFIE	CAU		CAU +SELFIE	CAU		CAU +SELFIE	CAU
Utilities (EQ–5D–5L)	0.63 (0.20)	0.58 (0.23)		0.69 (0.23)	0.63 (0.27)		0.75 (0.19)	0.65 (0.24)		0.69 (0.24)	0.68 (0.22)		0.71 (0.23)	0.67 (0.24)			
QALYs (EQ–5D–5L)																1.54 (.33)	1.43 (.37)
Self-esteem (RSES)	18.5 (3.8)	19.4 (3.7)		24.0 (5.4)	22.4 (4.9)		24.7 (5.7)	23.1 (5.7)		23.9 (6.3)	23.4 (5.4)		25.6 (6.7)	23.0 (5.7)			
Intervention costs																626	0
**Societal costs**																	
Healthcare costs^a^	877 (2255)	577 (724)		561 (917)	558 (1034)		409 (519)	302 (374)		580 (995)	410 (742)		373 (601)	457 (751)		2785 (3481)	2306 (2526)
Emergency care costs^b^	40 (131)	116 (537)		178 (1045)	161 (1119)		18 (138)	34 (176)		19 (93)	43 (199)		49 (149)	102 (640)		306 (1102)	458 (1738)
Medication costs	14 (64)	13 (58)		14 (64)	19 (90)		9 (20)	15 (103.2)		20 (30)	20 (138)		19 (31)	9 (17)		76 (162)	76 (334)
Productivity loss costs	313 (945)	218 (598)		148 (718)	172 (497)		316 (883)	320 (778)		389 (902)	249 (470)		343 (963)	138 (309)		1510 (2567)	1097 (1752)
**Total societal costs**	**1244 (2447)**	**923 (1181)**		**1964 (4474)**	**1984 (3768)**		**4910 (6707)**	**4395 (5870)**		**12782 (17194)**	**9173 (13124)**		**4652 (7889)**	**4089 (7246)**		**25471 (26812)**	**20507 (23392)**

*Abbreviations:* RSES = Rosenberg Self-esteem Scale (total scores range from 0 to 30 with higher scores indicating higher self-esteem); CAU = care as usual, QALY = quality-adjusted life year; EQ-5D-5L = EuroQol-5 Dimension Five Levels.

a Healthcare costs included costs of a general practitioner, general practitioner assistant in mental health care, psychologist, dietician, healthcare social worker, psychiatrist, substance use services, alternative care or semi-mural care, detailed in Table 1.

b Emergency care costs included costs of police at the door, police transport to a facility, an ambulance, crisis service use, ER care and psychiatric hospital care, detailed in Table 1.

### Cost-effectiveness and cost utility

Bootstrapping results showed that participants in the experimental condition (CAU + SELFIE) scored significantly higher on self-esteem at final follow-up than participants in the control condition (CAU only) (△Effects = 3.1, 95% bootstrap interval 1.7–4.4), with participants in the experimental condition also scoring higher on costs than participants in the control condition but this difference was nonsignificant (△Costs = €3,779, 95% bootstrap interval: €-1,841 to €9,844; see Table 4). Furthermore, QALYs were nonsignificantly higher in the experimental than the control condition (△QALY = 0.07, 95% bootstrap interval: −0.02 to 0.16).

**Table 4.** Bootstrapped results (bootstrap mean and 95% bootstrap interval) of the main analysis, sensitivity analyses, and subgroup analyses

Analysis	Participants (CAU + SELFIE/CAU)		Total costs € (CAU + SELFIE/CAU)		△Costs €^a^^i^	95% Bootstrap interval		△QALYs	95% Bootstrap interval		△Effects	95% Bootstrap interval		Probability cost-effective (%)^b^
**Base case** (societal perspective)	**85/89**		**25,157/19,673**		**3779**	**(−1841 to 9844)**		**0.07**	**(−0.02 to 0.16)**		**3.1**	(1.7 to 4.4)*		**26; 49%**
**Healthcare perspective**	85/89		15,670/13,234		1097	(−3078 to 5445)		^c^	^c^		^c^	^c^		56; 77%
**Sensitivity analyses**														
SEA1: Complete case analysis	37/41		16,442/15,919		1377	(−5164 to 8290)		0.12	(−0.04 to 0.27)		3.6	(1.4 to 5.8)*		62; 80%
SEA2: Without one-off intervention costs^d^	85/89		24,983/19,673		3605	(−2015 to 9670)		^c^	^c^		^c^	^c^		28; 50%
**Subgroup analyses**														
SGA1a: Recruited from secondary mental health services	43/44		24,775/22,314		−1311	(−7948 to 4920)		0.09	(−0.03 to 0.20)		2.8	(.7 to 4.8)*		78; 87%
SGA1b: Recruited from general population	42/45		25,482/17,159		8661	(−440 to 17,856)		0.04	(−0.08 to 0.17)		3.3	(1.5 to 5.0)*		7; 16%
SGA2a: In treatment^e^	71/70		21,837/25,076		1903	(−4388 to 8452)		0.09	(−0.01 to 0.19)		3.4	(1.8 to 4.9)*		49; 72%
SGA2b: Not in treatment	14/19		25,577/11,693		9353	(−2730 to 24,219)		0.02	(−0.18 to 0.26)		1.8	(−.6 to 4.1)		14; 23%
SGA3: Emotional abuse/neglect	64/63		27,844/20,251		5638	(−2049 to 13,474)		0.13	(0.02 to 0.24)*		3.1	(1.5 to 4.8)*		25; 55%
SGA4: Physical abuse/neglect	40/35		30,506/20,349		7924	(−1905 to 18,466)		0.05	(−0.11 to 0.21)		3.5	(1.5 to 5.4)*		13; 24%
SGA5: Sexual abuse	39/26		35,930/25,516		5562	(−6132 to 18,103)		0.10	(−0.08 to 0.27)		3.7	(1.9 to 6.1)*		32; 47%
SGA6: Bullying	82/74		26,545/19,976		4497	(−1684 to 11,142)		0.06	(−0.04 to 0.16)		3.2	(1.7 to 4.6)*		19; 38%
SGA7: Parental conflict	51/45		30,273/21,076		6336	(−3711 to 16,319)		0.08	(−0.06 to 0.21)		3.3	(1.5 to 5.2)*		19; 36%

*Abbreviations:* CAU = care as usual; SEA = sensitivity analysis, SGA = subgroup analysis; QALY = quality-adjusted life year.

a Incremental costs vary from the total cost difference between arms as they were based on regression model which includes correction for baseline.

b At the Dutch WTP-thresholds of €20,000, €50,000/QALY, respectively, for costs and QALYs.

c Identical to main analysis.

d Excludes the training of therapists and one-time application use costs, as detailed in Supplementary File 1.

e Receiving psychological treatment outside of the present study.

* If the 95% Bootstrap interval does not include zero, a significant difference is found at *a* = .05.

**Figure 2.**
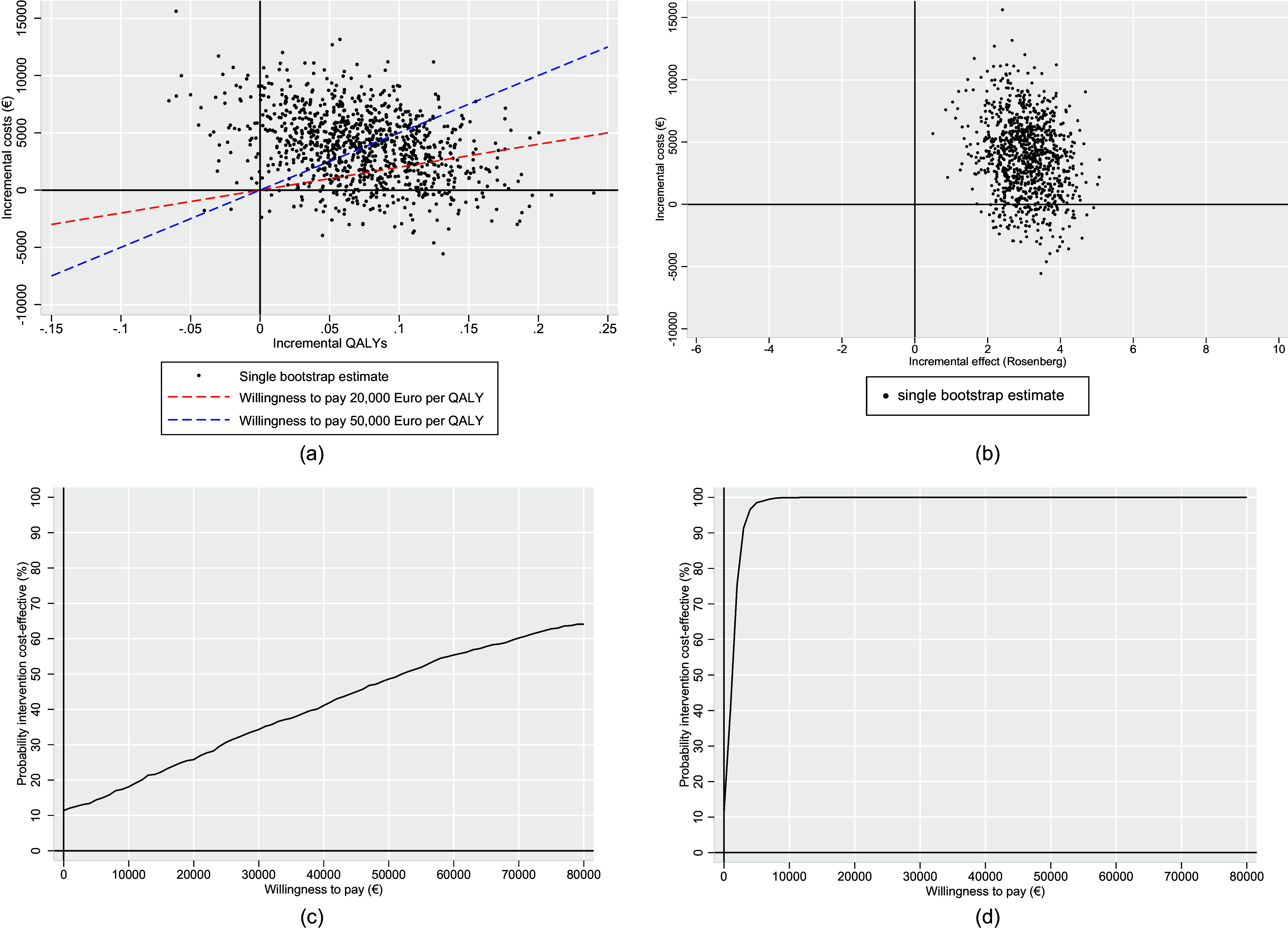
a. Incremental cost-utility plane (ICUP); b. Incremental cost-effectiveness plane (ICEP); c. Cost-utility acceptability curve (CUAC); d. Cost-effectiveness acceptability curve (CEAC).

In the main analysis, the ICER presented a cost of €3,779/3.1 = €1,219 per improved point of self-esteem on the RSES, and the ICUR presented a cost of €3,779/0.07 = €53,986 per QALY gained. The majority (84%) of the bootstrapped ICURs were in the northeast quadrant (see the ICUP in Figure 2a), indicating higher costs and higher QALYs. The same was found for the ICEP (Figure 2b). The majority (89%) of the bootstrapped ICERs were in the northeast quadrant as well, indicating higher costs and higher effects, measured with the RSES.

As seen in the CUAC (Figure 2c), the probability that the SELFIE intervention would be cost-effective was 26% at the WTP threshold of €20,000/QALY and 49% at the WTP threshold of €50,000/QALY. The CEAC (Figure 2d) shows that the probability that the SELFIE intervention would be cost-effective in terms of effects (RSES) was 99% starting at €5,000/RSES-unit and 100% starting at €12,000/RSES-unit.

### Sensitivity and subgroup analyses

From a healthcare perspective (i.e. excluding productivity loss costs), far smaller incremental costs (€1,097) and higher probabilities of cost-effectiveness were observed than in the main analysis (56% at €20,000/QALY; 77% at €50,000/QALY), with a cost of €1,097/0.07 = €15,671 per QALY gained, and €1,097/3.1 = €354 per improved point of self-esteem.

The complete case analysis yielded similar results to the main analysis in terms of bootstrap intervals (see Table 4). However, incremental costs in the complete case analysis were lower than in the main analysis (€1,377 vs. €3,779), incremental QALYs were higher (0.12 vs. 0.07), and the probability of the SELFIE intervention being cost-effective was much higher (62% at €20,000/QALY; 80% at €50,000/QALY).

Next, only excluding one-off intervention costs (i.e. cost-effectiveness if the application is already programmed and the practitioners already trained) yielded negligibly lower incremental costs (€3,604.91 vs. €3,779) and slightly higher cost-effectiveness probabilities (28% at €20,000/QALY; 50% at €50,000/QALY) than the main analysis.

Among the subgroups, the probability of being cost-effective was markedly higher than the main analysis for participants recruited from specialized mental health services (78% at €20,000/QALY; 87% at €50,000/QALY), in treatment (49% at €20,000/QALY; 72% at €50,000/QALY), and who experienced sexual abuse (32% at €20,000/QALY; 47% at €50,000/QALY).

## Discussion

### Principal findings

The current study moved beyond previous research by examining the cost-effectiveness and cost-utility of SELFIE, a cutting-edge transdiagnostic blended EMI for improving self-esteem in young people exposed to childhood adversity. To our knowledge, this is the first health-economic evaluation of an EMI delivered in youth. The base case (societal perspective, i.e., including all costs and all participants from both recruitment routes) showed a 26% probability of cost-effectiveness at the WTP threshold of €20,000/QALY and 49% at the WTP threshold of €50,000/QALY. In the exploratory sensitivity and subgroup analyses to assess robustness of results, these probabilities were 78% and 87%, respectively, for participants recruited from specialized mental health services and 56% and 77%, respectively, when adopting a healthcare perspective (that is excluding costs related to productivity loss).

### Comparison with findings from other studies

To date, reviews and syntheses of health-economic evaluations of mental health interventions for youth exposed to childhood adversity have generally focused on youth with (symptoms of) PTSD receiving trauma-focused (TF-CBT) and cognitive therapy for PTSD (CT-PTSD) (Mavranezouli, Megnin-Viggars, Trickey, et al., 2020; Spencer et al., 2023; von der Warth et al., 2020). Included studies indicated that TF-CBT was a cost-effective alternative to treatment as usual (Aas, Iversen, Holt, Ormhaug, & Jensen, 2018), no treatment (Gospodarevskaya & Segal, 2012), current practice (Mihalopoulos et al., 2015), and various PTSD-oriented therapy forms (Mavranezouli, Megnin-Viggars, Trickey, et al., 2020), and it was associated with lower costs for high-end mental health services (Greer, Grasso, Cohen, & Webb, 2014). CT-PTSD also outperformed care as usual (Shearer et al., 2018). One economic review focused on youth who experienced physical, sexual, or emotional child maltreatment (Macdonald et al., 2016). They found that the few existing cost-effectiveness studies were contradictory, inconclusive, or lacked fundamental aspects such as incremental analyses and benefits that were not sustained at 12-month follow-up.

Several methodological differences exist between the present study and previous health-economic evaluations for young people exposed to childhood adversity. First, the studies by Gospodarevskaya and Segal (2012) and Mihalopoulos et al. (2015) were modeled economic evaluations, not trial-based. Second, in previous studies, questionnaires other than the EQ-5D-5L were used to calculate utilities and QALYs, for example, the 16D (Aas et al., 2018), AQoL-4D (Gospodarevskaya & Segal, 2012; Mihalopoulos et al., 2015), and SDQ (Shearer et al., 2018). Third, in the present study, we adopted the societal perspective in the main analysis, whereas previous studies used a healthcare perspective only or did not explicitly report the perspective. Fourth, most studies included up to the age of 18 years (Aas et al., 2018; Greer et al., 2014; Mavranezouli, Megnin-Viggars, Trickey, et al., 2020), 17 years (Shearer et al., 2018), 16 years (and a separate group of adults) (Mihalopoulos et al., 2015), or starting at the age of 18 years (Mavranezouli, Megnin-Viggars, Grey, et al., 2020). It has frequently been shown that discontinuity of care at the age of 18 years is disadvantageous (Boonstra et al., 2024; Signorini et al., 2018) and shifting to interventions without harmful care transition necessitates health-economic evaluations of care across young personhood, as uniquely done in the present health-economic evaluation, the first known to us to have been conducted on a blended EMI for youth.

To the best of our knowledge, no economic studies to date have assessed blended EMIs for the prevention of detrimental later effects of childhood adversity and for intervention in young people. Among adults, one economic evaluation assessed an ESM-i for individuals with depression, finding a 46% probability of cost-effectiveness at WTP €50,000 (Simons et al., 2017), comparable to the 49% in the base case of the present study. In their study, as well as in the present study, interventions improved outcomes and showed reasonable cost-effectiveness. Together, these studies provide the first evidence on the cost-effectiveness of ESM-based monitoring and blended EMIs.

### Interpretation

In the base case, the SELFIE intervention exceeded the WTP threshold at the societal level. The sensitivity and subgroup analyses showed improved cost-effectiveness among those recruited from specialized mental health services, excluding the general population subgroup. In practice, the comparably lower costs and improved mental health among participants recruited from specialized mental health services indicate an improved ability to function (yielding higher productivity) and less need for care or medication (yielding lower care costs) as they received the SELFIE intervention, not seen in those recruited from the general population. It is tempting to speculate that this greater benefit in functioning may have resulted from mental health professionals’ selection of individuals for whom they expected beneficial effects for the SELFIE intervention, but, by definition, such effects would be balanced due to randomization to experimental and control condition. Replication and further insight into real-world translation are important next steps to clarify SELFIE’s health-economic benefit for routine practice.

Next, the healthcare perspective showed a high probability of cost-effectiveness as opposed to when costs of work absenteeism and suboptimal job performance were incorporated in the base case (societal perspective). It must be noted that questions in the TiC-P about work might have been unclear across conditions (e.g. interpreting working hours as actual hours worked or as contractual hours in case of prolonged partial illness). Moreover, many young adults aged 15–26 years are in education instead of, or next to, working. Hence, future health-economic research may consider measuring productivity loss at school (e.g. school absenteeism) and formally testing differences in employment status and contractual working hours between groups. Furthermore, police costs were included in both perspectives. Such official nonhealth sector costs can be included if relevant in the context of mental health and service use (Sittimart et al., 2024), which was deemed appropriate in the present study but may differ per context.

Participants who experienced childhood emotional neglect/abuse had the largest, and only significant, incremental effect on QALYs. The EQ-5D-5L, with which QALYs are calculated, contains domains such as anxiety/depression and usual activities that might be particularly affected by childhood emotional neglect/abuse. In previous research, emotional abuse was strongly associated with psychopathology, especially when combined with neglect (Korolevskaia & Yampolskaya, 2022). The association of emotional abuse with quality of life has been examined directly as well, albeit with a different instrument. For instance, emotional abuse has been found to be related to adult HRQoL, as measured by the Short Form 12 Health Survey (SF-12) (Piontek et al., 2021). It is unknown whether QALYs also improve more readily for other interventions for individuals exposed to childhood emotional neglect/abuse than for individuals exposed to other adversity types. To our knowledge, stratified analyses by type of adversity have not been conducted to date in health-economic evaluations of interventions for young people with prior exposure to childhood adversity. From the present results, it appears that the SELFIE intervention is especially cost-effective for young people who experienced childhood emotional neglect/abuse who were recruited from specialized mental health services.

### Methodological considerations

The current health-economic evaluation examined an innovative and accessible intervention method tailored to the age group of 12–26 years due to its digital channel for prevention and intervention in daily life. Furthermore, the study was established with expert advice by the authors of the guided self-help approach (De Neef, 2016) and assessed with youth advocates, refining the feasibility and acceptability of the EMI tasks for the target population. A methodological strength of the present study was the inclusion of a wide range of direct and indirect costs, including 12 types of mental health or specialized services, but also ambulance use, police at the door and/or police transport, productivity loss by missing work or reduced performance while at work, and medication use.

A limitation of the present study might be constrained generalizability due to the underrepresentation of young men, only one participant identifying as nonbinary, and few participants being of a migrant background or of an ethnic minority. In contrast, education level was evenly distributed with the highest representation for educational levels classified as ‘low’ or ‘middle’. Given the adherence and compliance, this suggests that the SELFIE intervention is accessible across young people of all educational levels. We should note, however, that differences in RSES or QALY gains were not statistically evaluated per educational level.

Second, another consideration is that the data on service and medication use in this study were based on self-report measures rather than on, for example, retrieved health record data from health authorities. Self-reporting might have introduced recall bias as participants could have imperfectly estimated their resource use, though this bias would have operated equally across experimental and control conditions, and the recall periods were short (4 weeks for service use and 2 weeks for medication use). Future studies might explore options to (partially) extract service use and medication use data externally.

Third, limitations exist for some of the measures used. The RSES generally does not have an empirically established minimally clinically important difference, meaning we cannot conclude how many young people reached such a cutoff. QALYs are the empirically established standard for health-economic analyses and should guide decision-making most strongly. Next, while an EQ-5D-Y-5 L (a young person’s version) exists, we decided to only use the EQ-5D-5L, to not divide age groups, and to compare across the study population. The EQ-5D-5L User Guide states that the age of 12–25 is an overlapping area in which both versions can be used (EuroQol Research Foundation, 2019). Nevertheless, the wording in the EQ-5D-5L was originally developed for adults and we cannot rule out the potential influence of this decision on the age-specific validity and sensitivity through, for example, reduced understanding or responsiveness. No participant indicated any difficulty understanding the questionnaire. Furthermore, utility values were based on the tariff from Versteegh et al. (2016) that was calculated from people aged 18 years or older. Hence, for those aged under 18 years in the present study, utilities could potentially have been under- or overestimated as age may influence valuation. No established youth tariffs existed for the 5-level EQ-5D or EQ-5D-Y at the time of analyzing.

Finally, not all potential financial costs could be measured, and thus the societal perspective was limited. For example, we did not measure time investment for parental care, transportation costs for on-site sessions, and informal care.

### Conclusion, policy implications and future research

The SELFIE intervention, a transdiagnostic blended EMI for young people with low self-esteem who have been exposed to childhood adversity, showed beneficial effects over time, and now improved cost-effectiveness for people receiving care from specialized mental health services, while it did not for those recruited from the general population. If replicated, and if real-world adherence is sufficiently equal, investment in SELFIE might particularly be worthwhile for delivery in routine public mental health care, especially for participants who experienced childhood emotional abuse/neglect. In the base case that included both the clinical and general population, cost-effectiveness was highly dependent on work productivity loss costs, showing good value for money from a healthcare perspective. Future health-economic evaluations of EMIs for youth should consider school absenteeism as a factor for productivity loss to obtain a fuller picture of the societal perspective, can include an even more varied population, and might contemplate new approaches that reflect quality of life more closely in daily life than using the standard utility approach with the EQ-5D-5L.

## Supporting information

10.1017/S003329172610395X.sm001 Boonstra et al. supplementary material Boonstra et al. supplementary material

## Supplementary material

The supplementary material for this article can be found at http://doi.org/10.1017/S003329172610395X.
