# Supplementary material for: Cost-utility and cost-effectiveness of SELFIE, a transdiagnostic ecological momentary intervention for improving self-esteem in youth aged 12–26 years exposed to childhood adversity: Findings from a multicenter randomized controlled trial
Source: Psychol Med. 2026 Apr 13;56:e103. doi: 10.1017/S003329172610395X (PMC13079239; doi:10.1017/S003329172610395X)
Supplement: Boonstra et al. supplementary material [file S003329172610395Xsup001.docx]

*Supplementary materials*

***Supplementary Table 1***

*Number and proportion of participants with complete endpoint data.*

|  | **Complete data N (%)** | | |
| --- | --- | --- | --- |
|  | **CAU+SELFIE (N=85)** | **CAU (N=89)** | **Total  (N=174)** |
| **Baseline** |  |  |  |
| Gender | 85 (100%) | 89 (100%) | 174 (100%) |
| Age | 85 (100%) | 89 (100%) | 174 (100%) |
| Level of education | 85 (100%) | 88 (99%) | 173 (99%) |
| Self-assigned ethnicity | 85 (100%) | 89 (100%) | 174 (100%) |
| TIC-P | 85 (100%) | 88 (99%) | 173 (99%) |
| Medication | 85 (100%) | 89 (100%) | 174 (100%) |
| EQ-5D-5L | 85 (100%) | 89 (100%) | 174 (100%) |
| RSES | 85 (100%) | 89 (100%) | 174 (100%) |
| **T1 (post-intervention)** |  |  |  |
| TIC-P | 71 (69%) | 80 (90%) | 151 (87%) |
| Medication | 71 (84%) | 77 (87%) | 148 (85%) |
| EQ-5D-5L | 71 (84%) | 80 (90%) | 151 (87%) |
| RSES | 74 (87%) | 81 (91%) | 155 (89%) |
| **T2 (6-month follow-up)** |  |  |  |
| TIC-P | 62 (73%) | 72 (81%) | 134 (77%) |
| Medication | 60 (71%) | 68 (76%) | 128 (74%) |
| EQ-5D-5L | 63 (74%) | 72 (81%) | 135 (78%) |
| RSES | 66 (78%) | 76 (85%) | 142 (82%) |
| **T3 (18-month follow-up)** |  |  |  |
| TIC-P | 49 (58%) | 57 (64%) | 106 (61%) |
| Medication | 46 (54%) | 51 (57%) | 97 (56%) |
| EQ-5D-5L | 49 (58%) | 57 (64%) | 106 (61%) |
| RSES | 55 (65%) | 65 (73%) | 120 (69%) |
| **T4 (24-month follow-up)** |  |  |  |
| TIC-P | 54 (64%) | 70 (79%) | 124 (71%) |
| Medication | 53 (62%) | 69 (78%) | 122 (70%) |
| EQ-5D-5L | 54 (64%) | 70 (79%) | 124 (71%) |
| RSES | 55 (65%) | 72 (81%) | 127 (73%) |
| *Abbreviations: CAU = Care as usual, RSES = Rosenberg Self-Esteem Scale, TIC-P = Trimbos/iMTA Questionnaire for Costs Associated with Psychiatric Illness.* | | | |

***Supplementary Table 2***

| *CHEERS 2022 Checklist* | | | |
| --- | --- | --- | --- |
| Topic | No. | Item | Location where item is reported |
| Title |  |  |  |
|  | 1 | Identify the study as an economic evaluation and specify the interventions being compared. | Title |
| Abstract |  |  |  |
|  | 2 | Provide a structured summary that highlights context, key methods, results, and alternative analyses. | Abstract |
| Introduction |  |  |  |
| Background and objectives | 3 | Give the context for the study, the study question, and its practical relevance for decision making in policy or practice. | Introduction |
| Methods |  |  |  |
| Health economic analysis plan | 4 | Indicate whether a health economic analysis plan was developed and where available. | Methods: Ethics statement and study design, Paragraph 1 |
| Study population | 5 | Describe characteristics of the study population (such as age range, demographics, socioeconomic, or clinical characteristics). | Methods: Participants + Table 2 |
| Setting and location | 6 | Provide relevant contextual information that may influence findings. | Methods: Experimental condition and control condition |
| Comparators | 7 | Describe the interventions or strategies being compared and why chosen. | Methods: Experimental condition and control condition |
| Perspective | 8 | State the perspective(s) adopted by the study and why chosen. | Methods: Analyses |
| Time horizon | 9 | State the time horizon for the study and why appropriate. | Methods: Main analyses |
| Discount rate | 10 | Report the discount rate(s) and reason chosen. | Methods: Cost calculations; Main analyses |
| Selection of outcomes | 11 | Describe what outcomes were used as the measure(s) of benefit(s) and harm(s). | Methods: Measures |
| Measurement of outcomes | 12 | Describe how outcomes used to capture benefit(s) and harm(s) were measured. | Methods: Measures; Analyses |
| Valuation of outcomes | 13 | Describe the population and methods used to measure and value outcomes. | Methods: Measures; Main analyses |
| Measurement and valuation of resources and costs | 14 | Describe how costs were valued. | Methods: Measures; Main analyses |
| Currency, price date, and conversion | 15 | Report the dates of the estimated resource quantities and unit costs, plus the currency and year of conversion. | Methods: Cost calculations + Table 1 |
| Rationale and description of model | 16 | If modelling is used, describe in detail and why used. Report if the model is publicly available and where it can be accessed. | Not applicable, non-modelling |
| Analytics and assumptions | 17 | Describe any methods for analysing or statistically transforming data, any extrapolation methods, and approaches for validating any model used. | Methods: Main analyses |
| Characterising heterogeneity | 18 | Describe any methods used for estimating how the results of the study vary for subgroups. | Methods: Main analyses; Sensitivity and subgroup analyses |
| Characterising distributional effects | 19 | Describe how impacts are distributed across different individuals or adjustments made to reflect priority populations. | Not applicable |
| Characterising uncertainty | 20 | Describe methods to characterise any sources of uncertainty in the analysis. | Methods: Main analyses; Sensitivity and subgroup analyses |
| Approach to engagement with patients and others affected by the study | 21 | Describe any approaches to engage patients or service recipients, the general public, communities, or stakeholders (such as clinicians or payers) in the design of the study. | Strengths and limitations |
| Results |  |  |  |
| Study parameters | 22 | Report all analytic inputs (such as values, ranges, references) including uncertainty or distributional assumptions. | Not applicable, non-modelling |
| Summary of main results | 23 | Report the mean values for the main categories of costs and outcomes of interest and summarise them in the most appropriate overall measure. | Results, Table 4+5 |
| Effect of uncertainty | 24 | Describe how uncertainty about analytic judgments, inputs, or projections affect findings. Report the effect of choice of discount rate and time horizon, if applicable. | Results: Cost-effectiveness and cost-utility; Sensitivity analyses and subgroup analyses + Table 5 + Fig.1-5 |
| Effect of engagement with patients and others affected by the study | 25 | Report on any difference patient/service recipient, general public, community, or stakeholder involvement made to the approach or findings of the study | Strengths and limitations |
| Discussion |  |  |  |
| Study findings, limitations, generalisability, and current knowledge | 26 | Report key findings, limitations, ethical or equity considerations not captured, and how these could affect patients, policy, or practice. | Discussion |
| Other relevant information |  |  |  |
| Source of funding | 27 | Describe how the study was funded and any role of the funder in the identification, design, conduct, and reporting of the analysis | End of manuscript |
| Conflicts of interest | 28 | Report authors conflicts of interest according to journal or International Committee of Medical Journal Editors requirements. | End of manuscript |

*From:*

Husereau, D., Drummond, M., Augustovski, F., de Bekker-Grob, E., Briggs, A. H., Carswell, C., Caulley,

L., Chaiyakunapruk, N., Greenberg, D., Loder, E., Mauskopf, J., Mullins, D., Petrou, S., Pwu, R., & Staniszewska, S. (2022a). Consolidated health economic evaluation reporting standards (CHEERS) 2022 Explanation and elaboration: a report of the ISPOR CHEERS II good practices task force. *Value in Health*, *25*(1), 10-31. <https://doi.org/10.1016/j.jval.2021.10.008>

Husereau, D., Drummond, M., Augustovski, F., de Bekker-Grob, E., Briggs, A. H., Carswell, C., Caulley,

L., Chaiyakunapruk, N., Greenberg, D., Loder, E., Mauskopf, J., Mullins, D., Petrou, S., Pwu, R., & Staniszewska, S. (2022b). Consolidated Health Economic Evaluation Reporting Standards 2022 (CHEERS 2022) statement: updated reporting guidance for health economic evaluations. *MDM Policy & Practice*, *7*(1). <https://doi.org/10.1177/23814683211061097>

***Supplementary File 1***

**SELFIE Intervention Costs**

The total SELFIE intervention costs were calculated per participant and were split into two main categories:

(1) *One-off costs*, and (2) *Participation costs*. These categories were combined to calculate the total intervention costs for the base case, while for *sensitivity analysis 2* only the second part was used: *the participation costs*.

1. One-off costs

| Standard unit price care “Psychologist, primary care” |  | Inflation factor |  | Standard inflation-adjusted unit price “Psychologist, primary care” |  | Average training hours |  | Number of therapists trained |  | Total training costs |
| --- | --- | --- | --- | --- | --- | --- | --- | --- | --- | --- |
| € 110,00 | * | 1,0209 | = | €112,30 | * | 4 | * | 18 | = | €8085,60 |

Eighteen therapists were trained to deliver the SELFIE intervention throughout a training that lasted on average four hours. The standard unit price of one hour of work by a psychologist in primary mental health services was derived from Hakkaart-van Roijen et al. (2024a) as it best represented the occupation of the therapists. Total costs of training were then calculated by multiplying that standard price with the inflation factor derived from Hakkaart-van Roijen et al. (2024b) projected to 2023. This outcome was then multiplied by the average number of training hours and the number of trained therapists.

| Total training costs *(n=18 therapists)* |  | One-off application costs |  | Total one-off costs |
| --- | --- | --- | --- | --- |
| €8085.60 | + | €6695.00 | = | €14,781 |

| Total one-off costs |  | Number of participants |  | Total one-off costs per participant |
| --- | --- | --- | --- | --- |
| €14,781 | / | 85 | = | €174.00 |

Next, one-off application costs of the intervention included the server rent for the study duration and developer time used for implementation such as costs to program the intervention into the application and to test and tailor the intervention before use. While programming, testing and tailoring were included, early-stage R&D costs (e.g. therapeutic concept development) were not. To arrive at the total price of the one-off costs, the total training costs of the eighteen therapists were added with the one-of application costs.

Next, total one-off costs were divided by the number of participants, which was n=85 (intention-to-treat principle), arriving at the cost per participant.

1. Participation costs

Participation costs consisted of application costs and therapist contact costs, the latter divided into face-to-face contacts and e-mail contacts. First, participation application costs were calculated.

| Per-person connection costs and costs of network traffic |  | Number of participants |  | Total participation application costs |
| --- | --- | --- | --- | --- |
| € 30.00 | * | 85 | = | €2588.00 |

Participation application costs included the per-person connection costs and costs of network traffic, which were multiplied by the number of participants to arrive at the total participation application costs.

| Standard inflation-adjusted unit price “Psychologist, primary care” |  | Hours of face-to-face contact |  | Number of participants |  | Total costs of face-to-face contacts |
| --- | --- | --- | --- | --- | --- | --- |
| €112.30 | * | 3 | * | 85 | = | €28,636 |

The first part of the contact costs were the face-to-face contacts, for which the standard price of a psychologist working in primary care was again derived from Hakkaart-van Roijen et al. (2024a) as it best represented the occupation of the therapists. This standard price was multiplied by the three hours of contact and the number of participants to derive the total costs of the face-to-face contacts.

| Standard inflation-adjusted unit price “Psychologist, primary care” |  | From one hour to ¼ hour |  | Price per e-mail contact |  | Number of e-mail contacts |  | Number of participants |  | Total costs of e-mail contacts |
| --- | --- | --- | --- | --- | --- | --- | --- | --- | --- | --- |
| €112.30 | / | 4 | = | €28.08 | * | 3 | * | 85 | = | €7,159 |

The second part of the therapist contact costs were the e-mail contacts. The standard price of an hour was divided by four to arrive at the ¼ hour of each e-mail contact and then multiplied by the three contacts and the 85 participants, resulting in the total costs of e-mail contacts.

| Total participation application costs |  | Total costs of face-to-face contacts |  | Total costs of  e-mail contacts |  | Total participation costs |
| --- | --- | --- | --- | --- | --- | --- |
| €2588.00 | + | €28,636 | + | €7,159 | = | €38,384 |

Next, the three total costs were added: the costs of the participation in the application, the face-to-face contacts and the e-mail contacts, together forming the total participation costs.

| Total participation costs |  | Number of participants |  | Total participation costs per participant |
| --- | --- | --- | --- | --- |
| €38,384 | / | 85 | = | €452.00 |

To display the total participation costs per participant, the total costs were divided by the 85 participants.

**Total intervention costs**

| Total one-off costs |  | Total participation costs |  | Total intervention costs | Number of participants |  |  | Total costs per participant |
| --- | --- | --- | --- | --- | --- | --- | --- | --- |
| €14,781 | + | €38,384 | = | €53,164 | / | 85 | = | €626.00 |

Finally, the total one-off costs and total participation costs were added together to arrive at the total intervention costs. These were divided by the number of participants to display the total costs per participant.

***Supplementary File 2***

| ***1. Amount of missing data*** | The amount of missing data for each variable and per occasion is detailed in Supplementary Table 1. All data (100%) was complete for all measures at baseline. For education level and TIC-P, data were missing for one person resulting in 99% completeness of data. The post-intervention completeness rate was 89% for the RSES, 87% for the TiC-P and EQ-5D-5L, and 85% for the medication checklist. At T4, this was 73%, 71%, and 70%, respectively. |
| --- | --- |
| ***2. Reasons for missingness*** | The reasons for being lost to follow-up are detailed in Figure 1, which include participants either being unreachable or having withdrawn from the study. Investigation into the missingness pattern revealed that the missing data mostly followed a monotone pattern, meaning participants with missing data at one time point tended to also have missing data at subsequent time points. Additionally, the follow-up outcome utility at six months significantly predicted missing total costs (*p*=.011), indicating a relationship between the outcome measurements and missing data. However, no baseline variables were found to predict missingness, suggesting that the missing data was not associated with other baseline variables in the study. |
| ***3. Consequences*** | Incremental effects in the key outcomes had the same directionality and the same outcomes were significant in both the complete and incomplete case analyses. No significant incremental effect was found for QALYs (0.07 in the base case and 0.12 in the complete case analysis) or costs (€1,377 for complete case analysis and €3,779 in the base case), while incremental effects (RSES) were significant (3.1 in the base case and 3.6 in the complete case analysis). |
| ***4. Method*** | Missing data were inspected, classified as missing-at-random, and handled by use of multiple imputation with 20 replications on the end-point level in terms of QALYs, RSES, and four cost categories (medication use, productivity loss, healthcare use, and emergency care use). |
| ***5. Software*** | All analyses were performed using STATA (Version 17.0, Standard Edition). The “mi impute” STATA command was used and the multiple imputation process involved chained equations using predictive mean matching (pmm) to impute the endpoint outcomes and it was stratified by the study arm. |
| ***6. Number of imputed datasets*** | A total of 20 imputed datasets were generated. |
| ***7. Imputation model*** | The following outcomes at T2 (post-intervention) and T3 (6 months), T4 (18 months), and T5 (24 months) were imputed with the multiple imputation model: RSES, EQ-5D-5L utility score, cost categories (healthcare costs, emergency care costs, medication costs, productivity loss costs).  The following baseline predictors were used: RSES, EQ-5D-5L utility score, healthcare costs, emergency care costs, medication costs, productivity loss costs, age (was group into categories of 12y-16y; 17y-21y; 22y-25y), sex, educational level, work, and ethnicity. Due to the very low prevalence of missing baseline data (see Supplementary Table 1; data completeness ranged from 99% to 100%), missing values were handled using mean imputation for continuous variables and mode imputation for categorical variables. |
| ***8. Derived variables*** | The subgroups of the Childhood Trauma Questionnaire were determined by using the moderate-severe cut-off scores posed by Bernstein and Fink (1998): >= 13 for Emotional Abuse; >= 10 for Physical Abuse; >= 15 for Emotional Neglect; and >= 10 for Physical Neglect and; >= 8 for Sexual Abuse. For parental conflict, measured with the interview measure of the Childhood Experience of Care and Abuse (CECA) (Bifulco et al., 1994), the cut-off was at >=3, meaning for frequency, “regularly” and “often” were classified at “yes”, and for severity, “severe” and “violent” were classified as “yes”. For bullying, measured with the Retrospective Bullying Questionnaire (Schäfer et al., 2004), frequency was dichotomized with a cut-off of >=3, meaning “frequently” and “constantly”, while for severity it was >=4, meaning “quite serious” and “extremely serious". No other recodes or transformations of variables were made to obtain the imputed bootstrapped results.  Bernstein D., Fink L. (1998). Childhood Trauma Questionnaire. A Retrospective Self-Report Questionnaire and Manual. San Antonio, The Psychological Corporation.  Bifulco, A., Brown, G. W., & Harris, T. O. (1994). Childhood Experience of Care and Abuse (CECA): a retrospective interview measure. *Journal of Child Psychology and Psychiatry*, *35*(8), 1419-1435. https://doi.org/10.1111/j.1469-7610.1994.tb01284.x  Schäfer, M., Korn, S., Smith, P. K., Hunter, S. C., Mora‐Merchán, J. A., Singer, M. M., & Van der Meulen, K. (2004). Lonely in the crowd: Recollections of bullying. *British Journal of Developmental Psychology*, *22*(3), 379-394. http://dx.doi.org/10.1348/0261510041552756 |
| ***9. Diagnostics*** | We checked the convergence of the regression models in all imputations by counting the number of times a model did not converge. During the multiple imputation procedure, convergence issues did not occur.  A data plausibility check was performed on the imputed values to ensure the imputation model did not generate unrealistic values. We checked the RSES, where all imputed values fell within the range of the scale, confirming their plausibility. For the utility scores, all imputed values were within the range of the observed values and adhered to the minimum and maximum bounds of the Dutch EQ-5D-5L tariff. Additionally, healthcare costs, emergency care costs, medication costs, productivity loss costs showed no negative values or values outside the observed ranges. Overall, the imputed values appeared realistic and consistent with both the observed data and the expected plausible ranges. |
| ***10. Pooling*** | Instead of pooling the estimates from the 20 imputed datasets, we employed a bootstrapping approach. This involved resampling the imputed datasets to calculate the incremental outcomes, allowing us to capture variability and uncertainty in the estimates. No explicit pooling or transformation of statistics was performed as per Rubin’s rules, but the bootstrapped results serve a comparable purpose. |
| ***11. Complete-case analysis*** | Both the incremental costs and incremental QALYs were non-significant in both the multiple imputation results (base case) and the complete-case analysis results. In both, incremental effects were significant, with a 3.1 in the base case and 3.6 in the complete case analysis. The probability of cost-effectiveness was much higher in the complete cases analysis (62% at WTP €20,000 and 80% at WTP €50,000) than in the base case (26% at WTP €20,000 and 49% at WTP €50,000), mostly due to differences in incremental costs (€1,377 for complete case analysis and €3,779 in the base case). Hence, only from the complete case analysis, cost-effectiveness would be established at WTP €50,000. |
| ***12. Sensitivity analysis*** | A sensitivity analysis has been conducted with an alternative handling missing data approach (complete-case analysis). However, no additional sensitivity analyses have been conducted departing from an alternative missing data mechanism. |

van Buuren, S. (2018). *Flexible imputation of missing data* (2^nd^ Edition). Chapman & Hall/CRC. Retrieved from: <https://stefvanbuuren.name/fimd/sec-reporting.html>
